# Evaluation of sequence hybridization for respiratory viruses using the Twist Bioscience Respiratory Virus Research panel and the OneCodex Respiratory Virus sequence analysis workflow

**DOI:** 10.1099/mgen.0.001103

**Published:** 2023-09-07

**Authors:** Natalia Kapel, Elizabeth Kalimeris, Sheila Lumley, Arun Decano, Gillian Rodger, Marcela Lopes Alves, Kate Dingle, Sarah Oakley, Lucinda Barrett, Sophie Barnett, Derrick Crook, David W. Eyre, Philippa C. Matthews, Teresa Street, Nicole Stoesser

**Affiliations:** ^1^​ Nuffield Department of Medicine, Oxford, UK; ^2^​ Oxford University Hospitals NHS Foundation Trust, Oxford, UK; ^3^​ NIHR Oxford Biomedical Research Centre, Oxford, UK; ^4^​ Big Data Institute, Nuffield Department of Public Health, Oxford, UK; ^5^​ Francis Crick Institute, London, UK

**Keywords:** bait capture, target enrichment, influenza, SARS-CoV-2, rhinovirus, diagnostics, sequencing

## Abstract

Respiratory viral infections are a major global clinical problem, and rapid, cheap, scalable and agnostic diagnostic tests that capture genome-level information on viral variation are urgently needed. Metagenomic approaches would be ideal, but remain currently limited in that much of the genetic content in respiratory samples is human, and amplifying and sequencing the viral/pathogen component in an unbiased manner is challenging. PCR-based tests, including those which detect multiple pathogens, are already widely used, but do not capture information on strain-level variation; tests with larger viral repertoires are also expensive on a per-test basis. One intermediate approach is the use of large panels of viral probes or ‘baits’, which target or ‘capture’ sequences representing complete genomes amongst several different common viral pathogens; these are then amplified, sequenced and analysed with a sequence analysis workflow. Here we evaluate one such commercial bait capture method (the Twist Bioscience Respiratory Virus Research Panel) and sequence analysis workflow (OneCodex), using control (simulated) and patient samples head-to-head with a validated multiplex PCR clinical diagnostic test (BioFire FilmArray). We highlight the limited sensitivity and specificity of the joint Twist Bioscience/OneCodex approach, which are further reduced by shortening workflow times and increasing sample throughput to reduce per-sample costs. These issues with performance may be driven by aspects of both the laboratory (e.g. capacity to enrich for viruses present in low numbers), bioinformatics methods used (e.g. a limited viral reference database) and thresholds adopted for calling a virus as present or absent. As a result, this workflow would require further optimization prior to any implementation for respiratory virus characterization in a routine diagnostic healthcare setting.

## Data Summary

Sequencing data have been uploaded to the ENA repository under BioProject accession number: PRJEB62137. Sample accession details are provided in Supplementary dataset S1, available with the online version of this article. The authors confirm all supporting data, code and protocols have been provided within the article or through supplementary data files. Supplementary datasets are available in the Microbiology Society’s data repository Figshare account: https://doi.org/10.6084/m9.figshare.23804091 [1].

**Impact Statement**

Most chest infections are caused by ~20 common viruses. Quickly, accurately and cheaply diagnosing which viruses cause chest infections remains challenging. This is because most tests either look for only one or two specific targets (e.g. flu, COVID-19), or are expensive. Several new approaches have been developed recently, including a process which uses probes on samples to enrich for viral genetic material, and then sequences this genetic material to understand what type and what strain of virus is present (this is known as ‘bait capture sequencing’). However, there are few studies that have characterized how these methods work on samples that are positive for viruses other than SARS-CoV-2. We therefore undertook a study to understand how one of these targeted bait capture methods, the Twist Bioscience Respiratory Virus Research Panel and OneCodex sequence analysis workflow, worked on samples where we knew what and how much virus the sample contained, and real-life human samples. Our results show that this method is not able to reliably detect target viruses when used in a cost-effective manner, and that the method also calls other closely related viruses as present when they are not. Further optimization of this approach, or alternative approaches, would be needed for diagnostic use in healthcare.

## Introduction

Respiratory tract infections are a common global cause of morbidity, hospital admission and mortality [2], and accurate respiratory virus diagnostics are key to patient management and effective Infection Prevention and Control strategies to limit transmission, particularly in healthcare and institutional settings. PCR-based tests are regularly used for diagnosis, although tests targeting multiple pathogens rapidly and accurately, such as the BioFire FilmArray Respiratory Panels, are relatively expensive on a per-test basis. In addition, the SARS-CoV-2 pandemic has demonstrated the value of genomic approaches to detecting and tracking viral infection and variants, enabling surveillance of transmission and evolution in response to treatments and vaccines; conventional PCR assays do not capture this information [3]. The recent resurgence of other respiratory viruses such as respiratory syncytial virus (RSV) and influenza, and concerns around the ongoing zoonotic risk posed by avian influenza, have highlighted that sequencing-based approaches could also have utility for wider respiratory virus diagnostics and surveillance.

However, direct sequencing of respiratory samples remains challenging, as these samples also contain large numbers of human cells as well as pathogen particles, and the proportion of human reads generated frequently dwarfs the number of pathogen-associated sequences: this approach therefore lacks sensitivity and resolution at the whole genome level. As a result, several target enrichment approaches have been pursued, including tiling multiplex PCRs and sequence hybridization approaches. Whilst the former are fast, straightforward and highly scalable, they are problematic in the context of viral evolution given the intolerance of the process for primer-sequence mismatches, and this results in sequencing dropouts [4]. Targeted bait capture approaches, on the other hand, reportedly tolerate greater divergence between target sequences and the biotinylated probes used that are complementary to the targeted nucleic acids [5], to some extent because these are longer than primers. They are also theoretically capable of identifying a larger and more diverse set of viral pathogens in a single assay using large panels of probes, although some focus on singular targets, in particular SARS-CoV-2 [5].

Of the several commercially available bait capture methods for the detection of multiple respiratory viruses, two current predominant ones are the Twist Bioscience Respiratory Virus Research (RVR) panel (Catalogue no. 103068; ~41 000 120 nt probes targeting 29 viruses/strains; Table S1), and the Illumina Respiratory Virus Oligos Panel (Catalogue no. 20044311; ~7800 probes targeting 41 viruses/strains). Previous work investigating the performance of SARS-CoV-2-specific and respiratory virus bait capture panels in detecting SARS-CoV-2 has suggested that the greatest enrichment of targets was achieved with Twist Bioscience panels, with greater post-enrichment fragment sizes and lower PCR duplication ratios than the Illumina panels, but less efficient relative human target depletion [5]. However, limited wider evaluations of these methods for the sequence-based detection of a range of respiratory viruses have been undertaken.

We therefore focused our resources on a pilot evaluation of the Twist Bioscience RVR panel and an associated sequence analysis workflow developed by OneCodex against multiple different respiratory virus targets, first by evaluating performance on synthetic control sequences (influenza, rhinovirus and SARS-CoV-2), second by testing paired nasopharyngeal and saliva samples that had been analysed using our current diagnostic standard, the BioFire FilmArray Respiratory Panel 2.1, and finally by investigating the possibility of multiplexing larger numbers of samples and shortening the hybridization time to maximize throughput and minimize per-sample cost.

## Methods

### Synthetic viral controls and simulated samples with synthetic viral spike-ins

We purchased three non-infectious synthetic viral controls from Twist Bioscience for our simulated sample experiments, namely: SARS-CoV-2 [catalogue no: 104533, Delta Strain, B.1.617.2, ssRNA (+ sense), 29 000 bases], influenza A H3N2 [catalogue no: 103002, ssRNA (− sense), 13 627 bases] and rhinovirus 89 [catalogue no: 103006, ssRNA (+ sense), 7152 bases]. These controls are generated through the synthesis of single or multiple DNA fragments transcribed into ssRNA. Each control reflects >99.9 % of the reference viral genome and is supplied at a concentration of 1 million copies µl^–1^.

As simulated samples, the following duplicate dilutions of each Twist viral control were spiked into human universal RNA (Agilent; 1 µg µl^–1^) and tested: 10^2^, 10^3^ and 10^6^ viral genome copies µl^–1^. Negative water and un-spiked universal RNA controls were included in triplicate. Eight samples were multiplexed in each pool for testing as per the manufacturer’s instructions, with 24 samples on a sequencing run.

### Patient samples

Paired nasopharyngeal and saliva (oropharyngeal) samples were collected from consenting patients with respiratory symptoms presenting to the Oxford University Hospital NHS Foundation Trust (Oxford, UK), and processed using the BioFire FilmArray Respiratory Panel 2.1 (BioFire RP2.1, in accordance with the manufacturer’s instructions) which detects 19 viral and four bacterial respiratory pathogens (Table S1).

### Sample nucleic acid extraction and quantification

All patient samples were extracted using the KingFisher Flex platform (Thermo Fisher Scientific) and the MagMAX Viral/Pathogen Nucleic Acid Kit (catalogue no: A42352; Thermo Fisher Scientific). Nucleic acid in all samples was quantified using the Qubit fluorometer (Thermo Fisher Scientific), and fragment length distributions were evaluated using the TapeStation System (Agilent) at multiple stages of the workflow, in accordance with the Twist Bioscience protocols.

### Twist RVR target enrichment processes

For sample preparation, we followed the Twist Bioscience ‘Dilution of Nucleic Acid Samples and optional Synthetic Controls’ and ‘cDNA Synthesis and Perform Quality control (QC)’ sections of the ‘Twist Total Nucleic Acids Library Preparation Kit for Viral Pathogen Detection and Characterisation’ protocol. For library preparation the ‘Library Preparation EF 2.0 with Enzymatic Fragmentation and Twist Universal Adapter System’ protocol was used, and for target enrichment the ‘Twist Target Enrichment Protocol’ was used (Fig. S1). For the initial evaluation of the simulated samples and all of the clinical samples we followed the manufacturer’s recommendation to multiplex eight samples per pool.

### Twist RVR fast hybridization and increased multiplexing process

With the recommendation to multiplex eight samples per pool and use the standard hybridization workflow, the consumables cost per sample is currently ~£140 per sample and the turnaround time >2 days – both major limitations for implementation as a diagnostic assay. Twist Biosciences have released a Fast Hybridization kit and protocol, designed to shorten the hybridization time from 16 h to somewhere between 15 min and 4 h. A single previous study had investigated the multiplexing of 20 instead of eight samples per pool to investigate respiratory virus co-infection in SARS-CoV-2-positive patients [6]; we therefore trialled a similar approach with the faster hybridization time to see if we could achieve similar results to the standard workflow/hybridization time with a shorter and cheaper workflow.

To enable a direct comparison with the 8-plex/standard hybridization run, for the 20-plex/fast hybridization experiment we used the simulated duplicate viral spike-in samples that had been stored at the end of the library preparation stage at −20 °C (prior to target enrichment, as per the manufacturer’s instructions), including the duplicate dilutions of influenza A H3N2, rhinovirus 89 and SARS-CoV-2 at 10^2^, 10^3^ and 10^6^ viral genome copies µl^–1^, alongside two of the negative water controls (r1, r2).

### Sequencing

Sample preparation for sequencing for all experiments was conducted following the ‘MiSeq System Denature and Dilute Libraries Guide’ protocol (Document no: 15039740 v10) for the MiSeq Reagent Kit v3 (150-cycle) chemistry. Sequencing was carried out on an Illumina MiSeq, generating 75 bp paired-end reads.

### Sequence data analysis

We used the OneCodex platform (https://www.onecodex.com) for sequencing data analysis, as a pragmatic approach considering that these workflows might be used in diagnostic laboratories without specialist bioinformatic support, and OneCodex vouchers are supplied with the Twist Bioscience RVR panel. OneCodex is a cloud-based tool with specific workflows for various commercial target enrichment panels, including the Twist Bioscience RVR panel being assessed here. The user interface and data upload are intuitive and straightforward, and output reports include numerical and graphical results.

**Fig. 1.**
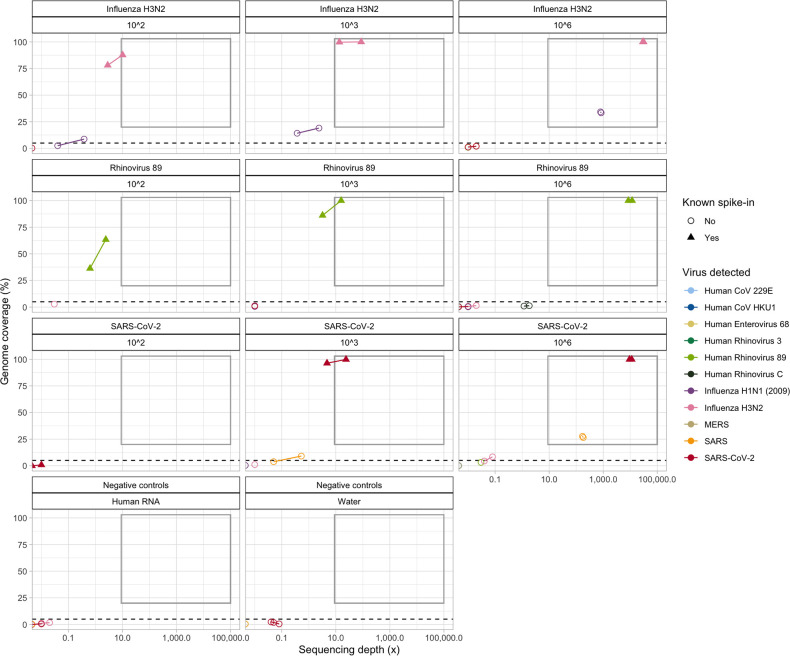
OneCodex sequencing depth (×) and genome coverage estimates (%) for each spike-in sample (replicates of influenza H3N2, rhinovirus 89 and SARS-CoV-2 delta), at three different concentrations (10^2^, 10^3^ and 10^6^ viral genome copies µl^–1^), with eight samples multiplexed. ‘Genome coverage’ denotes the proportion of the relevant viral reference genome covered by at least one sequence; ‘sequencing depth’ denotes the mean sequencing depth across the entire reference genome. Points within the grey box reflect samples which meet the combined OneCodex coverage/depth thresholds used to call a virus ‘detected’. All points above the dashed line but not in the grey box reflect samples that would meet the OneCodex coverage threshold used to call viral presence as ‘indeterminate’. Experiments on the virus dilution series were carried out in duplicate (**r1, r2**), and on the negative controls in triplicate (water and the universal human RNA used as the background for the spiked samples; **r1, r2, r3**). Replicate samples (where both samples had evidence of viral sequence detected) are joined by a line. Note the log scale on the *x*-axis.

To run the analysis in OneCodex, individual sequencing fastq files were uploaded manually to the OneCodex portal and the ‘Twist Respiratory Virus’ workflow (released 9 February 2020) in the ‘Run Analyses’ section was implemented as per the Users’ Guide at: https://docs.onecodex.com/en/articles/4256891-getting-started-with-the-twist-respiratory-virus-research-panel (this also includes an output report as an example). From the output reports for each run the coverage and depth values were extracted for the analysis represented in Fig. 1 (values summarized in Supplementary Dataset 2).

Although the details of the OneCodex workflow are proprietary, the developers state it uses sequence alignment to map reads to a database of reference sequences made up of those viruses that are included in the Twist Bioscience RVR panel (https://docs.onecodex.com/en/articles/4648676-bioinformatics-details-of-the-twist-viral-panels). The mapping is done using minimap2 (https://github.com/lh3/minimap2) with default alignment settings, and the built-in preset for aligning short input reads. Given homology between some viruses in the panel, multi-mapping is allowed for by retaining all secondary alignments of equal quality to the primary alignment. Subsequently, sequencing artefacts are filtered out of the sample based on the relative frequency of unique *k*-mers in the sample, a step which aims to minimize sequencing or reference genome artefacts without affecting low-abundance or low-confidence matches. Mean sequencing depth across the entire reference genome (‘depth’), fraction of the reference covered by at least one read (‘coverage’) and cumulative sequence identity (‘identity’) are then calculated. The OneCodex workflow stipulates that viruses are characterized as ‘detected’ if ≥20 % coverage of the genome is achieved with an overall average sequencing depth of ≥10×, or ‘indeterminate/possibly detected’ if ≥5 % coverage of the genome is achieved (irrespective of sequencing depth). All other viruses are noted to be ‘not detected’. It is important to note that this bioinformatics approach is designed to target the specific reference respiratory virus sequences present in the Twist Bioscience RVR panel (see Table S1), and that the inclusion of a restricted subset of viral sequences in the reference database may therefore result in matches to other members of the same viral family (e.g. diverse rhinoviruses) remaining unreported.

For the patient samples, where samples were tested using the BioFire RP2.1 and the Twist RVR/OneCodex workflow, we used a stringent approach for comparison defining the tests as ‘strictly concordant’ only if the virus type was considered ‘detected’ by the Twist RVR test (i.e. both the OneCodex genome coverage and sequencing depth thresholds were met) and these matched the BioFire results exactly.

### Statistical analysis

We used Spearman’s rank correlation coefficient to evaluate the strength of the correlation between sequencing depth and coverage.

## Results

### Testing on dilution series of control viruses spiked into human universal RNA (8-plex, 24 samples per sequencing run)

Across all three synthetic virus controls and spike-in concentrations, genome coverage for the spiked viral control was very strongly positively correlated with sequencing depth, as expected (*R*=0.94, *P*<0.001; Fig. 1). Genome coverage of 100 % at >1000× depth was achieved for all replicates with a spike-in of 10^6^ viral genome copies µl^–1^, and genome coverage of >75 % at >3× depth (average depth across the reference genome) for all replicates with a spike-in of 10^3^ viral genome copies µl^–1^. At 10^2^ viral genome copies µl^–1^, detection was much more variable, with the best coverage and depth achieved for influenza A H3N2 followed by rhinovirus, but only very low coverage and depth for SARS-CoV-2 (Fig. 1).

Specificity of sequencing read assignations was an issue, with hits to other viruses within the same family as the known target spike-in clearly apparent for influenza A H3N2 (with influenza A H1N1) and for SARS-CoV-2 (with SARS); no such issues were seen for rhinovirus 89 using the specified thresholds of detection (Fig. 1).

Sequencing reads mapping to viral reference sequences were present in five out of six negative controls, but all of these were below the OneCodex thresholds for calling detection (Fig. 1, Supplementary dataset S2). However, the sequences identified were all matches to either influenza A H3N2 or SARS-CoV reference sequences, consistent with low levels of cross-contamination of the synthetic sequences that were being used in the experiment (Fig. 1, Supplementary dataset S2).

### Testing on patient samples that had been tested using the BioFire FilmArray Respiratory Panel 2.1

Ten paired nasopharyngeal and saliva samples from ten unique patients and a single nasopharyngeal sample from an eleventh patient which had been tested on the BioFire RP2.1 panel were available for testing using the Twist RVR panel (*n*=21 samples). These included samples positive by BioFire for rhinovirus/enterovirus (*n*=8), parainfluenza 3 (*n*=7), adenovirus (*n*=5), SARS-CoV-2 (*n*=2) and human coronavirus NL63 (*n*=2); five samples were positive for more than one virus type and two samples were negative (Table 1).

**Table 1.** Viral detection in clinical samples by BioFire RP2.1 versus the Twist RVR panel Tests were defined as strictly concordant (‘yes’) where the virus type was considered ‘detected’ by the Twist RVR test (i.e. both OneCodex genome coverage and sequencing depth thresholds were met) and these matched the BioFire results exactly (‘indeterminate’ results were not considered in this definition). The BioFire RP2.1 test does not distinguish between rhinovirus and enterovirus. It also does not detect human bocavirus and so this was not considered in the concordance comparisons.

Patient study no.	Sample type	Virus type detected by BioFire RP2.1	Virus type ‘detected’ by Twist RVR (needs to meet both thresholds)		Virus type ‘indeterminate’ by Twist RVR	Tests strictly concordant?
			≥20 % genome coverage	≥10× sequencing depth	≥5 %, <20 % genome coverage	
RKX-0155	Nasopharyngeal s-0477	Rhinovirus/enterovirus	–	Rhinovirus 89	Rhinovirus 89	No
	Saliva s-0476	Rhinovirus/enterovirus	–	Rhinovirus 89	Human bocavirus 1	No
RKX-0164	Nasopharyngeal s-0471	Rhinovirus/enterovirus	–	Rhinovirus 89 Rhinovirus C	–	No
	Saliva s-0470	Rhinovirus/enterovirus	–	–	–	No
RKX-0176	Nasopharyngeal s-0365	–	–	–	–	Yes
	Saliva s-0364	–	–	–	Human bocavirus 1	Yes
RKX-0178	Nasopharyngeal s-0345	Adenovirus	–	Adenovirus type 7 Adenovirus 14 Adenovirus B1 Adenovirus E	Adenovirus E	No
	Saliva s-0344	Adenovirus	–	Adenovirus B1 Adenovirus E	Adenovirus E	No
RKX-0179	Nasopharyngeal s-0338	Rhinovirus/enterovirus Parainfluenza 3 virus	Parainfluenza 3 virus	Parainfluenza 3 virus	–	No
	Saliva s-0339	Parainfluenza 3 virus	Parainfluenza 3 virus	Parainfluenza 3 virus	–	Yes
RKX-0190	Nasopharyngeal s-0385	Parainfluenza 3 virus	Parainfluenza 3 virus	Parainfluenza 3 virus	–	Yes
	Saliva s-0384	Parainfluenza 3 virus	–	–	–	No
RKX-0208	Nasopharyngeal s-0368	Parainfluenza 3 virus	Parainfluenza 3 virus	Parainfluenza 3 virus	Human bocavirus 1	Yes
	Saliva s-0369	Parainfluenza 3 virus	Parainfluenza 3 virus	Parainfluenza 3 virus	Human bocavirus 1	Yes
RKX-0230	Nasopharyngeal s-0379	SARS-CoV-2	SARS-CoV-2	SARS-CoV-2	SARS-CoV-1	Yes
	Saliva s-0378	SARS-CoV-2	SARS-CoV-2	SARS-CoV-2	SARS-CoV-1	Yes
RKX-0241	Nasopharyngeal s-0302	Adenovirus	–	–	–	No
	Saliva s-0303	Adenovirus Rhinovirus/enterovirus	–	Adenovirus type 7 Adenovirus 14 Adenovirus B1 Adenovirus E	Adenovirus type 7 Adenovirus B1 Adenovirus E	No
RKX-0244	Nasopharyngeal s-0483	Rhinovirus/enterovirus Coronavirus NL63	Coronavirus NL63	–	–	No
	Saliva s-0482	Rhinovirus/enterovirus Coronavirus NL63	Coronavirus NL63	Coronavirus NL63 Enterovirus 68	–	No
RKX-0203	Nasopharyngeal	Adenovirus Parainfluenza 3	Human bocavirus 1	Human bocavirus 1	Parainfluenza 3 virus	No

Strict concordance of the Biofire RP2.1 and the Twist RVR panel results on patient samples was poor [8/21 (38 %) evaluations], although there were examples of overlaps where the RVR panel give an ‘indeterminate’ result for a pathogen detected by the BioFire RP2.1 assay (e.g. nasopharyngeal sample for RKX-0155, nasopharyngeal sample for RKX-0203; Table 1). Issues with specificity of the Twist RVR results as seen in the simulated samples were again demonstrated in the clinical samples – for example for patient RKX-0230 who was SARS-CoV-2 positive on both nasopharyngeal and saliva samples and the corresponding Twist results showed this as being detected, but with an additional ‘indeterminate’ detection call for SARS(-CoV-1). Similarly, for all the cases where the RVR panel identified adenovirus sequences, there were usually matches to multiple adenovirus references. Bocavirus signatures were observed using the RVR panel in 4/21 (19 %) samples. A more detailed breakdown of results for each patient is presented in the Supplementary Material (Fig. S2; Supplementary dataset S3).

### Multiplexing 20 simulated samples and using a fast hybridization approach

**Fig. 2.**
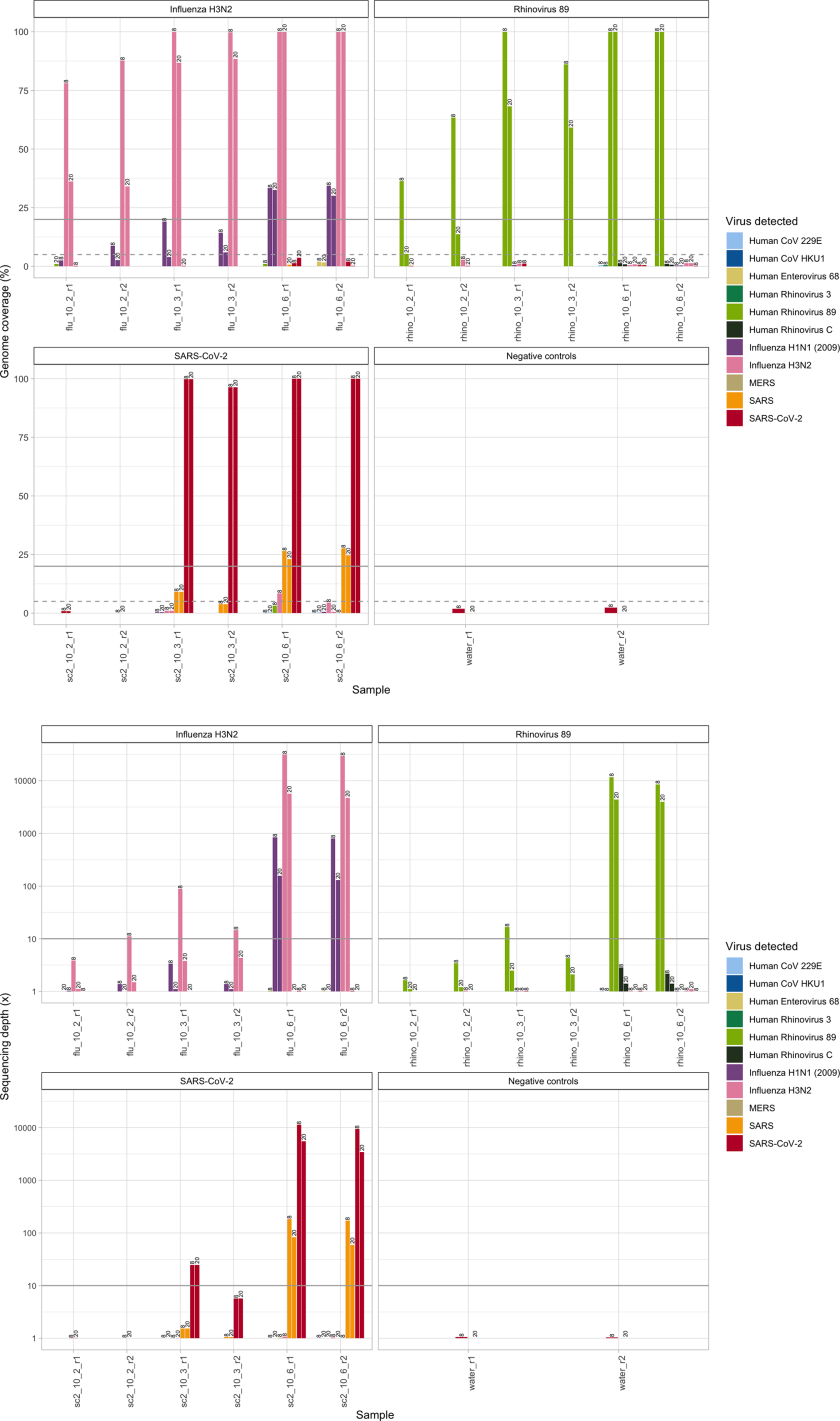
OneCodex sequencing depth (×) and genome coverage estimates (%) for each spike-in sample (replicates of influenza H3N2, rhinovirus 89 and SARS-CoV-2 delta), at three different concentrations (10^2^, 10^3^ and 10^6^ viral genome copies µl^–1^), for an 8-plexed reaction with the standard hybridization workflow and a 20-plexed reaction with the fast hybridization workflow. Results are presented for the OneCodex genome coverage (top) and average sequencing depth (bottom) outputs. Bold lines represent the thresholds required for ‘detected’ calls (i.e. both ≥20 % coverage of the genome and a sequencing depth of ≥10×) and dashed line the coverage value for ‘indeterminate’ calls (≥5 % coverage of the genome is achieved, irrespective of sequencing depth). Replicates are denoted ‘r1’ and ‘r2’; the workflow in the experiment is denoted by the number at the top of the bars. Note the log scale on the *y*-axis for the sequencing depth plot.

Whilst overall the results were broadly similar to pooling eight samples and using a longer hybridization time, genome coverage and sequencing depth values were lower for the two replicate dilutions <10^6^ viral genome copies µl^–1^, suggesting that shortening workflows by reducing the hybridization time to 2 h and multiplexing larger numbers of samples would limit assay sensitivity for samples with lower concentrations of virus (Fig. 2; Supplementary dataset S2).

## Discussion

Although bait capture methods appear to be an attractive approach to developing a diagnostic capable of generating strain-level information on multiple common respiratory pathogens, we encountered several problems in using the Twist RVR panel in a pragmatic and relatively ‘off-the-shelf’ way. The standard workflow is lengthy and time-consuming (2–3 days), and even with the fast hybridization approach, the shortest workflow time would be 1–1.5 days. Typical limits-of-detection for respiratory viral real-time PCR assays (e.g. SARS-CoV-2) are reportedly 10–500 copies µl^–1^ [7], a viral concentration at which we achieved incomplete reference genome coverage in our experiments with simulated samples across the three evaluated virus types, and only variably met sequencing depth thresholds. This problem became more marked when using shorter hybridization times and multiplexing more samples. Specificity was also a problem, with incorrect hits above the threshold for calling a virus ‘detected’ noted in both simulated and real clinical samples, when using the BioFire RP2.1 panel as the reference standard. For example, in simulated samples of SARS-CoV-2 at >10^6^ copiesreal-time PCR assays, SARS-CoV-1 was also ‘detected’, and in the SARS-CoV-2-positive clinical sample that was evaluated, SARS-CoV-1 was called as ‘indeterminate’. Similar issues were seen in distinguishing between influenza A H3N2 and H1N1 strains, and in adenovirus targets. The reported capacity of the Twist RVR panel to characterize infections at the strain level was therefore not supported in this evaluation.

There are few other published data evaluating the performance of the Twist RVR panel on either simulated or clinical samples; the major focus of these studies has been on SARS-CoV-2-positive samples [5, 6]. One study has investigated the rate of viral co-infections with SARS-CoV-2, but results were not reported at the viral strain level, and no evaluation was undertaken on simulated samples where the ground truth was known [6]. That study also investigated the sensitivity and precision of nucleotide-level variants detected from consensus Twist sequences when compared with amplicon whole genome sequencing data as the truth set, with single nucleotide variants identified correctly in 42/48 (88 %) of samples evaluated.

There were multiple limitations with our study. First, this was a pilot evaluation, limited by the available resources to cover the costs of the assay; as a result we undertook only a limited number of replicates and did not evaluate the full range of 10-fold dilutions for each of the simulated viral samples. Second, we used the recommended bioinformatics platform bundled with this test (OneCodex), which is a commercial product with a proprietary pipeline; we did not try and develop our own bioinformatics approach to characterizing reads and strain-level variability. Given the challenges that the workflow had in identifying strain-level variation, we did not pursue an investigation of sequence-level variation in greater detail, in order to evaluate, for example, whether it would be possible to accurately characterize resistance markers typical of antiviral resistance (e.g. for influenza A), or identify transmission events. Finally, although there are other commercially available bait capture assays for respiratory viruses, we did not consider those here, again due to resource limitations.

The impact of some of the challenges we identified on generating more sensitive and accurate results over a clinically meaningful timeframe could potentially be overcome – for example by developing automated workflows relying on robotics, or by optimizing bioinformatic workflows to try and account for some of the problems noted with specificity. However, it seems likely that the cost per sample, turnaround time and accuracy of the current test and recommended bioinformatics approach would preclude this assay and workflow being implementable for respiratory virus characterization in a routine diagnostic healthcare setting without significant further optimization.

## Supplementary Data

Supplementary material 1
