# Supplementary material for: Evaluation of sequence hybridization for respiratory viruses using the Twist Bioscience Respiratory Virus Research panel and the OneCodex Respiratory Virus sequence analysis workflow
Source: Microb Genom. 2023 Sep 7;9(9):001103. doi: 10.1099/mgen.0.001103 (PMC10569729; doi:10.1099/mgen.0.001103)

Supplementary information - Sequence hybridisation for respiratory viruses using the Twist Bioscience Respiratory panel

Table S1. Pathogens targeted by the various tests used in this study.

**Assay**	**Viral targets**	**Bacterial targets**
BioFire Film Array Respiratory Panel 2.1	Adenovirus<br>Coronavirus 229E<br>Coronavirus HKU1<br>Coronavirus NL63<br>Coronavirus OC43<br>Severe Acute Respiratory Syndrome Coronavirus 2 (SARS-CoV-2) Human Metapneumovirus<br>Human Rhinovirus/Enterovirus<br>Influenza A, including subtypes<br>H1, H3 and H1-2009<br>Influenza B<br>Parainfluenza Virus 1<br>Parainfluenza Virus 2<br>Parainfluenza Virus 3<br>Parainfluenza Virus 4<br>Respiratory Syncytial Virus	*Bordetella parapertussis*<br>*Bordetella pertussis*<br>*Chlamydia pneumoniae*<br>*Mycoplasma pneumoniae*
Twist Biosciences Respiratory Virus Research panel (and NCBI sequence accession numbers as per the Twist Biosciences "NGS Target Enrichment of Viral Pathogens using Twist Respiratory Virus Research Panel" Application note, Rev2.0, 12/Jul/2021; https://www.twistbioscience.com/resources/application-note/ngs-target-enrichment-viral-pathogens-using-twist-respiratory-virus)	Human adenovirus 14 (JN032132)<br>Human adenovirus B1 (NC_011203.1)<br>Human adenovirus E (NC_003266)<br>Human adenovirus type 7<br>(AC_000018)<br>Human bocavirus 1 (MG953830.1)<br>Human metapneumovirus<br>(NC_039199.1)<br>Human coronavirus 229E<br>(NC_002645.1)<br>Human coronavirus HKU1<br>(NC_006577.2)<br>Human coronavirus NL63<br>(NC_005831.2)<br>Human coronavirus OC43<br>(NC_006213.1)<br>Human enterovirus 68 (NC_038308.1)<br>SARS<br>(NC_004718.3)<br>SARS-CoV-2	-

	(NC_004718.3)<br>MERS<br>(JX869059.2)<br>Measles<br>(NC_001498.1)<br>Mumps<br>(NC_002200.1)<br>Rubella<br>(NC_001545.2)<br>Influenza B<br>(NC_002211,<br>NC_002204,<br>NC_002210,<br>NC_002209,<br>NC_002208,<br>NC_002207,<br>NC_002206,<br>NC_002205)<br>Influenza H1N1 (2009)<br>(NC_026432,<br>NC_026431,<br>NC_026434,<br>NC_026436,<br>NC_026433,<br>NC_026437,<br>NC_026435,<br>NC_026438)<br>Influenza H3N2<br>(NC_007369,<br>NC_007373,<br>NC_007372,<br>NC_007371,<br>NC_007366,<br>NC_007368,<br>NC_007367,<br>NC_007370)<br>Human rhinovirus 3<br>(NC_038312.1)<br>Human rhinovirus 89<br>(NC_001617.1)<br>Human rhinovirus C<br>(NC_009996.1)<br>Human parainfluenza virus 1<br>(NC_003461.1)<br>Human parainfluenza virus 3<br>(NC_001796.2)<br>Human parainfluenza virus 4<br>(NC_021928.1)<br>Human rubulavirus (parainfluenza virus 2)<br>(NC_003443.1)<br>Respiratory syncytial virus (A)<br>(NC_001803.1)<br>Respiratory syncytial virus (B) (NC_001781)	

Figure S1. Schematic of the sample processing workflow for the Twist RVR panel.
Image created using Biorender.com.

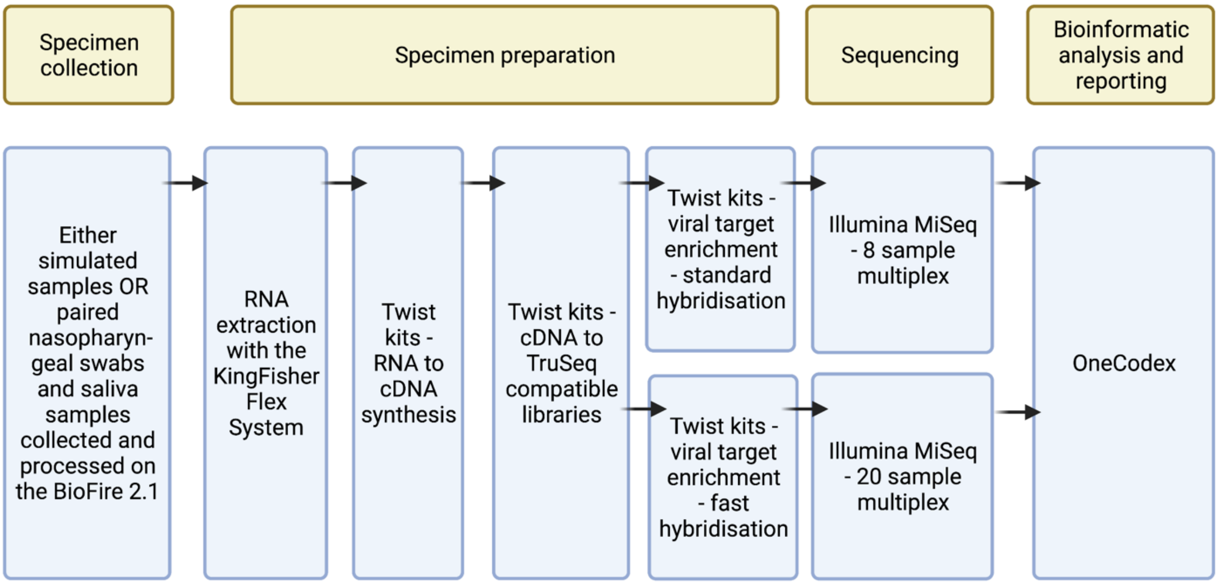

Figure S2. BioFire RP2.1 and Twist RVR results for each participant reflected as genome coverage (left panels) and sequencing depth plots (right panels). The BioFire RP2.1 panel is a non-quantitative test, and therefore all positives are reflected as bars reaching 100% on the y-axis in both panels.

Patient RKX-0155

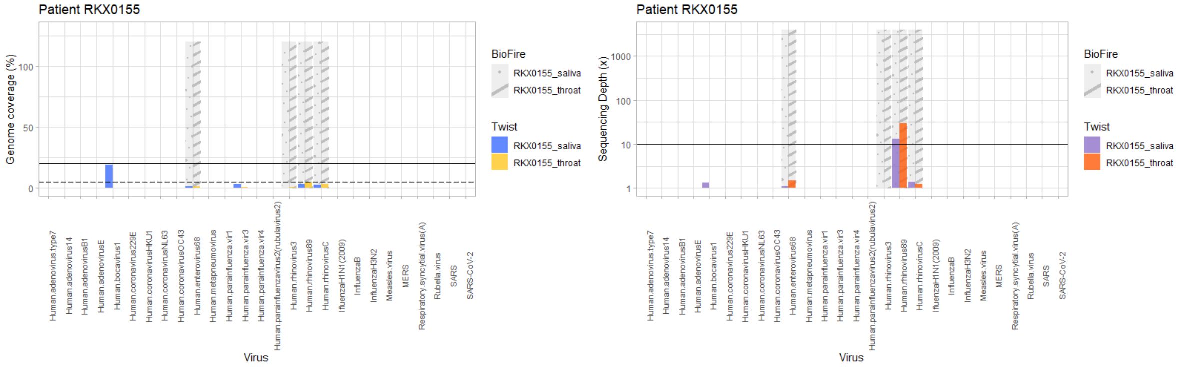

Patient RKX-0164

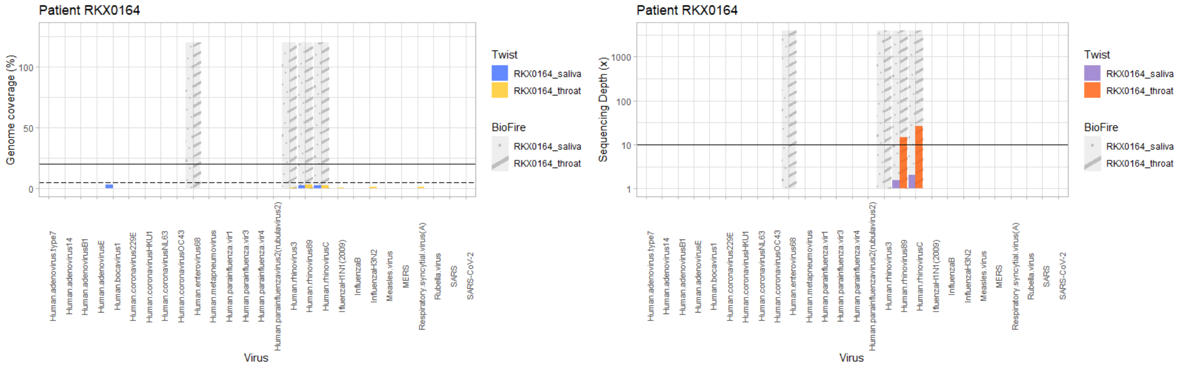

Patient RKX-0176

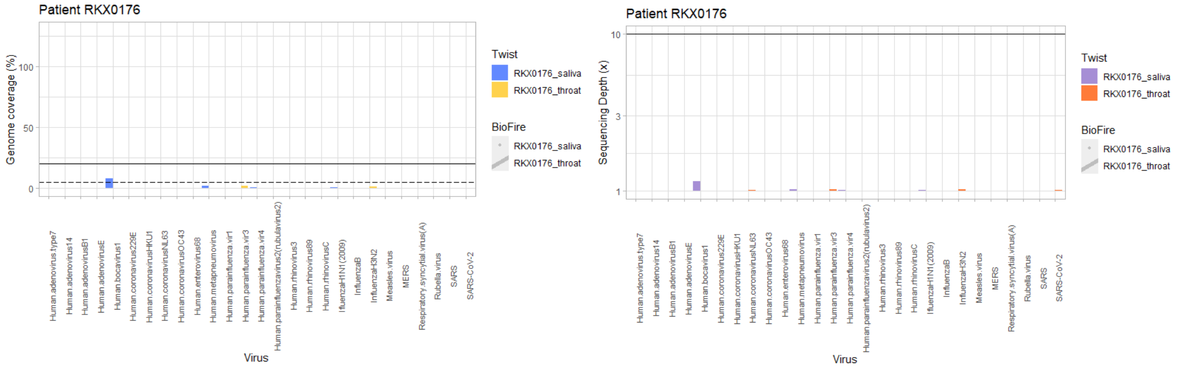

Patient RKX-0178

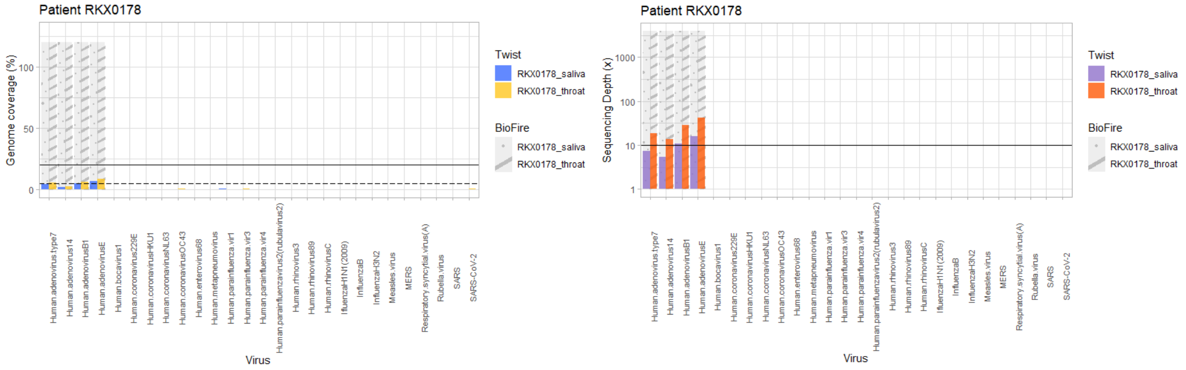

Patient RKX-0179

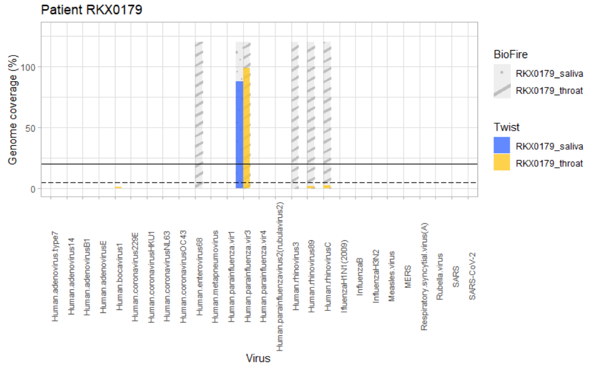

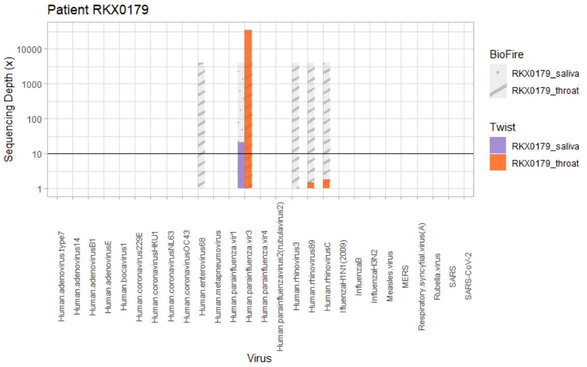

Patient RKX-0190

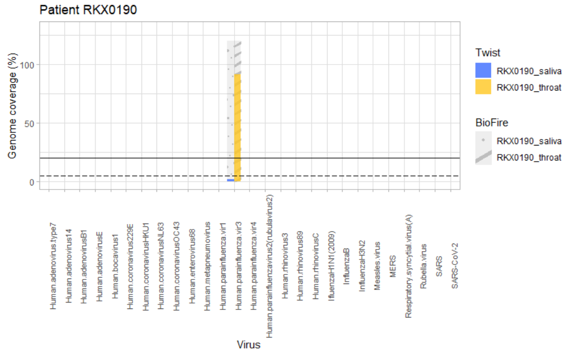

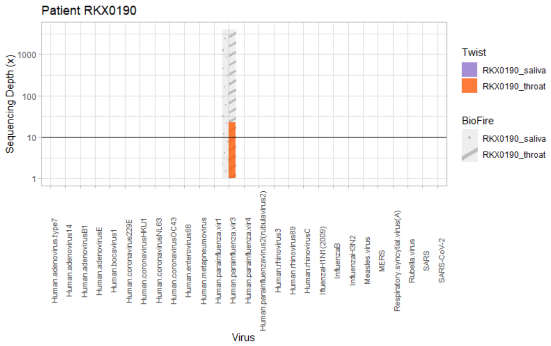

Patient RKX-0203

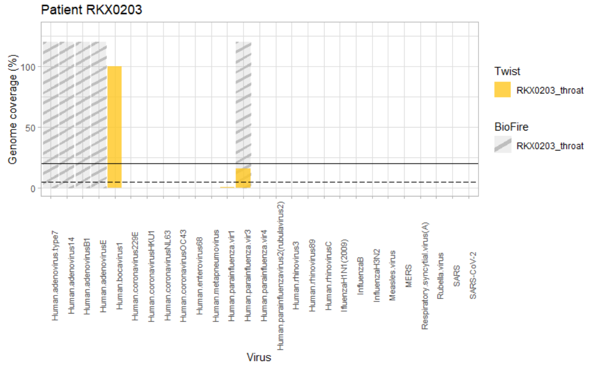

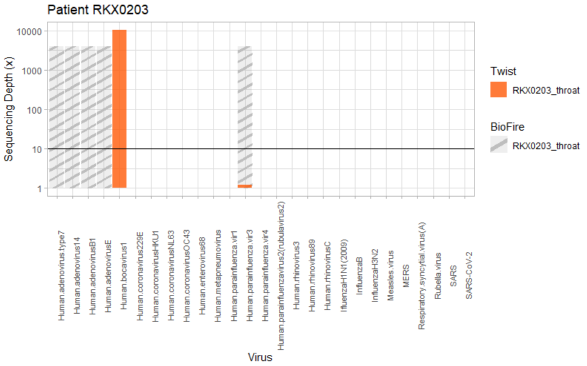

Patient RKX-0208

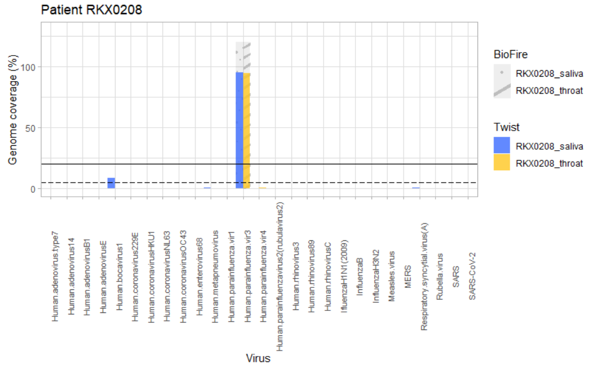

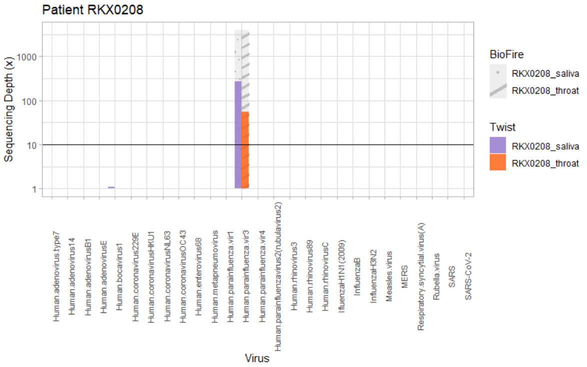

Patient RKX-0230

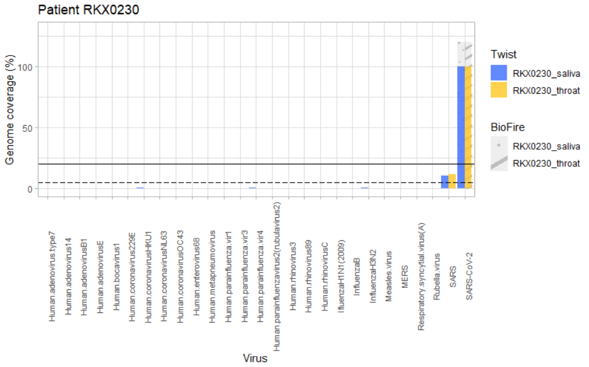

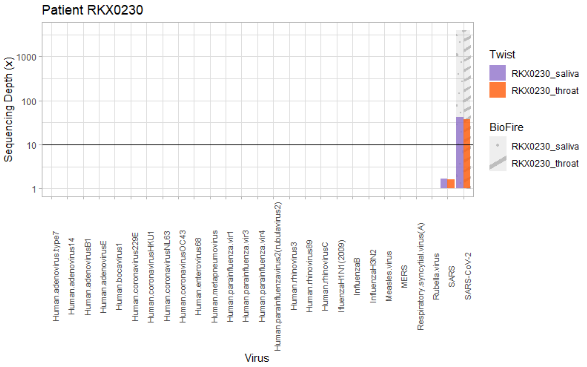

Patient RKX-041

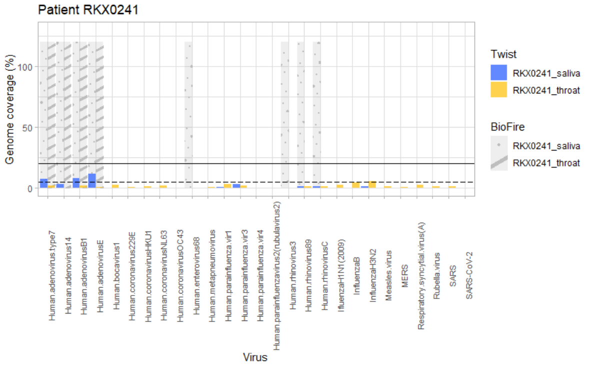

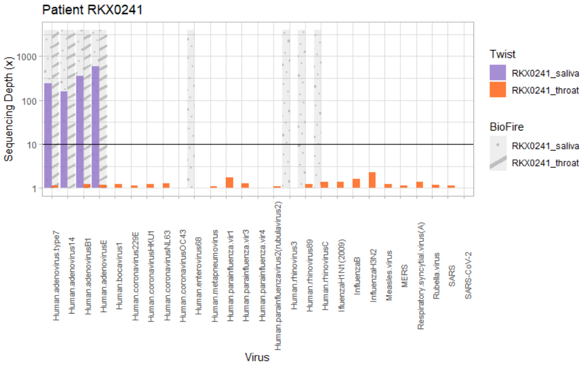

Patient RKX-0244

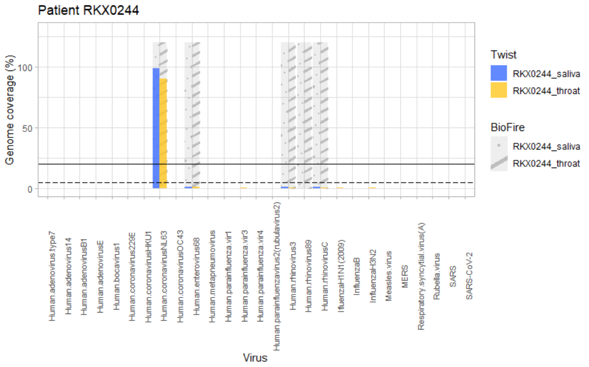

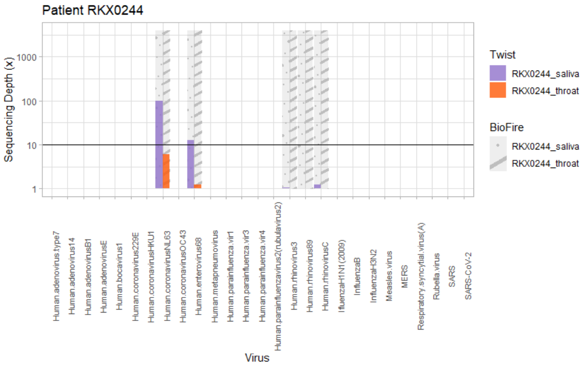

Supplement: Supplementary material 1 [file mgen-9-1103-s001.pdf]
